# Autumn leaf color brightness of Japanese alpine vegetation is projected to decrease under future climate change

**DOI:** 10.1038/s41598-025-14547-2

**Published:** 2025-08-08

**Authors:** Dai Koide, Reiko Ide, Hiroyuki Oguma, Keisuke Suzuki, Haruka Ohashi, Yuji Kominami

**Affiliations:** 1 National Institute for Environmental Studies, 16-2, Onogawa, Tsukuba, Ibaraki 305-8506 Japan; 2 Shinshu University, 3-1-1, Asahi, Matsumoto, Nagano, 390-8621 Japan; 3 Forestry and Forest Products Research Institute, 1 Matsunosato, Tsukuba, Ibaraki 305-8687 Japan

**Keywords:** Global climate model, Linear mixed model, Visible atmospherically resistant index, Repeated camera observation, Green-up day, *Sorbus Matsumurana*, Ecology, Plant sciences, Climate sciences, Ecology

## Abstract

Autumn leaf coloring is an essential cultural ecosystem service, but mechanisms of color brightness have scarcely been focused on, even though the autumn color crisis (leaf fall without coloring) has been reported in Japanese alpine shrubs. To approach the mechanisms, we analyzed the relationship between brightness and environmental conditions and projected possible future changes. Autumn color brightness was measured by repeated camera observations at three Japanese alpine sites. Environmental factors were analyzed using linear mixed model comparisons. Results showed earlier green-up timing was related to a duller autumn leaf color, possibly because a prolonged leaf period creates older leaves and reduces physiological vitality in autumn, affecting anthocyanin biosynthesis. Green-up timing based on snowmelt day and degree-day-based prediction of snowmelt day predicted earlier snowmelt and green-up in the future, with decreases in autumn color brightness. The amount of color reduction varied among climate models and scenarios, from a decline of several percent under MRI-CGCM3 RCP 2.6 to around 15% under MIROC5 RCP8.5 by the end of this century. Projected reductions in autumn color brightness could have an economic impact on tourism, and it could also be linked to modifications in material cycles and the ecosystem’s productivity.

**Supplementary Information:**

The online version contains supplementary material available at 10.1038/s41598-025-14547-2.

## Introduction

Autumn in temperate to sub-boreal regions in the Northern Hemisphere is marked by red, yellow, orange, and brown leaves. This autumn leaf coloring is an important cultural ecosystem service^1,2^ having commercial importance for tourism industries^3,4^ by creating beautiful landscapes full of color^5,6^. Many websites report observed or projected autumn leaf vistas (e.g., https://smokymountains.com/fall-foliage-map/, accessed on 2025/5/7). The timing of this event is reported to be described by several climatic drivers such as temperature^7–9^ day length, especially at high latitudes^10,11^ and drought^12^. This timing has economic importance when it aligns with holiday seasons or calendar-based festivals^13^. But the phenological timing is changing with climate change^14^ leading to potential changes in economic income^15^.

Aside from a culturally important ecosystem service, autumn leaf coloring is also an important marker of ecosystem functional processes. The phenological productive period (from leaf expansion to senescence) strongly drives above-ground productivity^16,17^. Leaf color pigments also support productivity through physiological protection for photosynthetic systems from several stress factors^18–20^. Such above-ground productivity directly affects below-ground activities through the quantity and quality of litterfall, affecting the rate of decomposition^21,22^ and root exudates^23^. These factors link ecosystem-scale functions such as productivity and the nitrogen and carbon balances^24,25^ suggesting a need to understand detailed mechanisms in autumn leaf coloring.

However, in addition to the limited reports on autumn phenology compared to spring^26^ previous studies on autumn leaf coloring have three limitations. Firstly, they have focused on timing (phenology), ignoring color brightness, despite its primal human appeal^5,6^. Autumn reds and yellows are due to the accumulation of anthocyanins and carotenoids, with a simultaneous reduction of chlorophylls^27^. Temperature and solar radiation determine the physiological response of these pigments and the brightness of autumn leaf color^28–30^. Stress factors such as solar radiation, drought, nutrient deficiency, salinity, and pests and diseases also affect pigment dynamics^19,31,32^. These reports, however, derive mainly from laboratory experiments or individual observations, so the narrow spatiotemporal scale is the second limitation, suggesting the need for a broader ecological-scale analysis. Which climatic factor or stress condition is important for local autumn color brightness is unclear at broad spatial scales, though there are a lot of broad-scale reports on phenological autumn color timings^33,34^. Future climate change could cause both increases and decreases in autumn leaf color brightness, but we found no reference to these in our review. The third limitation is that previous works have been focused mainly on temperate trees, with little attention to alpine vegetation^35^. Human habitation is concentrated in the temperate zone, so autumn leaf coloration there is important, but alpine autumn scenery is also important, especially for tourism. It is reported that autumn leaf coloring in Japanese alpine vegetation is eventually disappearing in some sites and years^35^ so an approach to the mechanisms underlying alpine autumn color brightness is necessary both academically and socially.

The aims of this paper are to clarify the relationship between autumn leaf color brightness of alpine vegetation and environmental conditions, and to project possible future changes in brightness in Japanese alpine vegetation. We used repeated camera observations and climate datasets to construct a linear mixed model to predict autumn color brightness. Using the model and future climate models, we projected future changes in the autumn color brightness on a national scale. We posed two key questions: Which of the analyzed environmental variables is most strongly correlated with the autumn leaf color brightness in Japanese alpine vegetation? Will autumn leaf color brightness increase or decrease under future climate warming scenarios?

## Results

### Green-up day affected autumn leaf color brightness

**Fig. 1:**
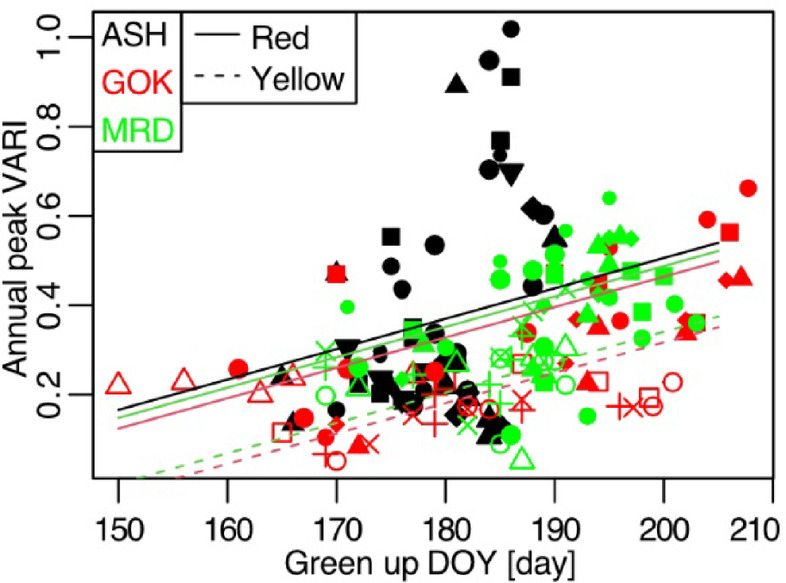
Response lines of the best model. Different symbols represent areas of interest (AOIs), and filled symbols mean red coloring. Note that the ASH site did not contain yellow coloring AOIs. Regression lines by the linear mixed model 5 in Table 1 are plotted.

After model comparisons between the response (i.e., annual maximum autumn color brightness, which was indexed by the visible atmospherically resistant index (VARI)) and predictor variables (Supplementary Table S1), the model containing green-up day and July mean temperature best explained the annual maximum VARI (model 1, Table 1). The green-up day had a higher standardized parameter value (0.069 ± 0.012, estimated parameter by model 1 and its standard error, *n* = 160) than July mean temperature (0.056 ± 0.013), and it was selected in all top 10 models, suggesting that it is the most important variable for autumn leaf color brightness in our sites. On the other hand, July temperature was selected in only 3 of the top 10 models. VARI showed vague responses to other explanatory variables (Supplementary Fig. S1), suggesting low explanatory power. Considering relatively weaker effects of the other variables and following wide uncertainty in future projections, we used the green-up day only model (model 5) to predict future changes in autumn leaf color brightness, though the coefficient of determination was the lowest in model 5 among the top 10 models (Table 1). As the green-up day and annual peak VARI had a positive correlation (Fig. 1), early green-up years would have duller autumn leaf colors according to this model.

**Table 1:** The best 10 model structures and their performances.

Model	Structure	AIC	*R* ^2^
1	Gup + Temp_7	−130.3	0.51
2	Gup + Prec_8	−126.5	0.51
3	Gup + Temp_7 + GSR_8	−124.1	0.51
4	Gup + Prec_8 + Sun_8	−122.8	0.53
5	Gup	−122.2	0.47
6	Gup + Temp_7 + Sun_8	−121.5	0.51
7	Gup + Prec_8 + Sun_7	−120.1	0.52
8	Gup + GSR_8 + Prec_8	−118.9	0.51
9	Gup + GSR_7 + Prec_8	−117.5	0.51
10	Gup + Sun_7	−116.0	0.48

“_7”, July; “_8”, August. Gup, green-up day; Temp, mean temperature; Tmax, maximum temperature; Prec, precipitation; GSR, global solar radiation; Sun, sunshine duration. AIC, Akaike’s information criteria.

### Future VARI reduction through earlier snowmelt and green-up

**Fig. 2:**
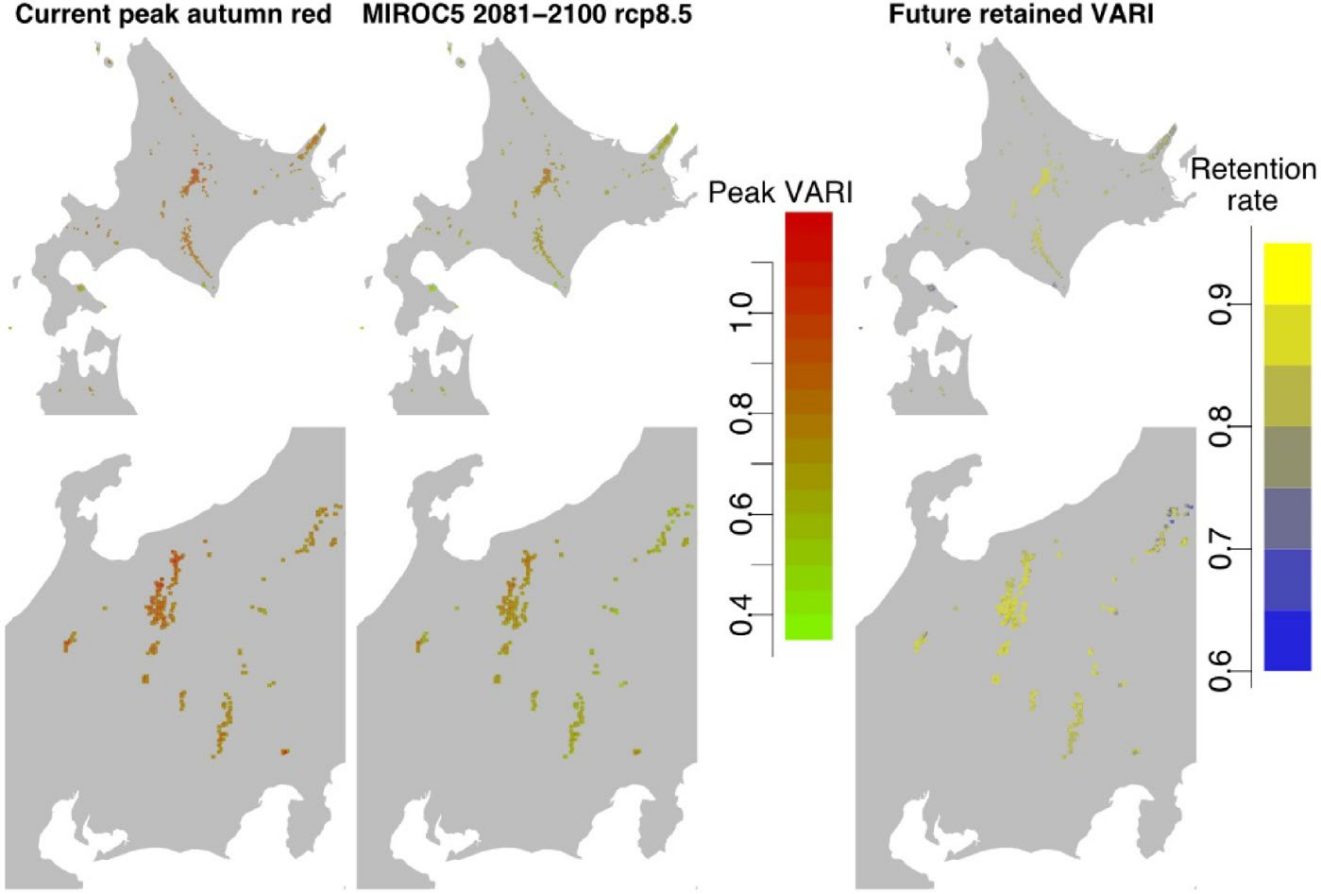
Maps of current (1981–2000) and future (2081–2100) projected autumn leaf color brightness and its future retained rate. Differences among GCMs and RCPs are shown in Supplementary Fig. S4.

**Fig. 3:**
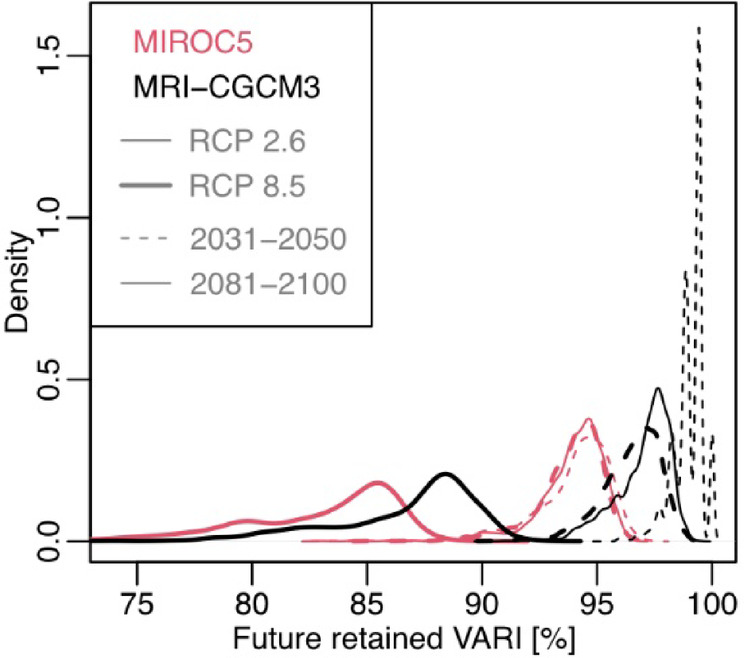
Density plot of future retention of autumn leaf color brightness among GCMs and RCP scenarios.

To predict future changes in autumn leaf color brightness, we predicted future green-up days using the correlation between green-up days and snow-melt days in our research site (Supplementary Fig. S2) and a degree-day-based snow projection model (details are in the Materials and methods part). Using the predicted future green-up days, we predicted future autumn color brightness. Under future warming and subsequent advancement of snowmelt and green-up days (Supplementary Figs. S2, S3), alpine VARI was projected to decrease nationally (Fig. 2, Supplementary Fig. S4). The decrease varied widely among GCMs and RCPs, from several percentage points under MRI-CGCM3 RCP2.6 2031–2050 to ~ 15% under MIROC5 RCP8.5 2081–2100 (Fig. 3). In some cells, it reached 25% reduction under MIROC5 RCP8.5 2081–2100 (Figs. 2 and 3). The degree of reduction was consistent with the degree of warming (Supplementary Fig. S3), suggesting greater VARI reduction under severe warming scenario. MRI-CGCM3 RCP2.6 2031–2050 predicted an increase of < 1 °C in annual mean temperature, but MIROC5 RCP8.5 2081–2100 predicted around 5 °C. Warmer conditions would advance snowmelt and green-up days, creating duller autumn leaf colors.

### Vulnerable areas

**Fig. 4:**
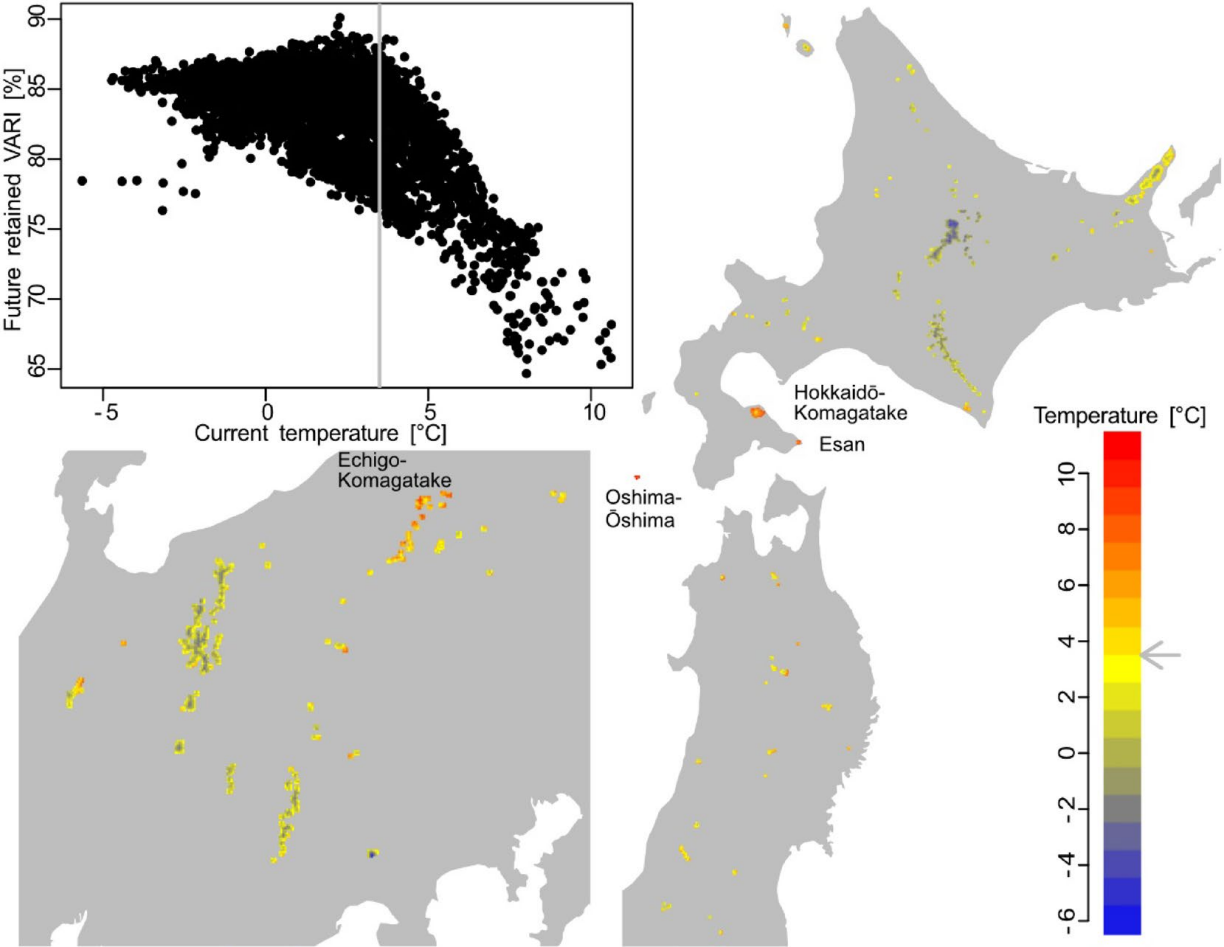
The future retention of autumn leaf color brightness (VARI) and the current temperature distribution map. The scatter plot between future retained VARI (Fig. 2) and current annual mean temperature (1981–2000) for the alpine vegetation cells shows that the future VARI decline is evident in current high temperature cells (i.e., temperature > 3.5 °C). Future VARI change was projected using the MIROC5 RCP8.5 scenario in 2081–2100.

Spatial differences in future VARI changes were related to current temperature conditions: sites with greater VARI reduction appeared in warmer areas, especially those whose current mean temperature is > 3.5 °C (Fig. 4). Such warmer sites at lower elevations represent populations of alpine plants that are vulnerable not only in their distribution^36^ but also in autumn leaf color brightness. Vulnerable alpine vegetation was predicted in southern Hokkaido, such as on Mt. Hokkaidō-Komakgatake, Mt. Esan, and Oshima-Ōshima (Figs. 2 and 4). Another typically vulnerable region was predicted in the northern part of the central region, such as in the Echigo-Komagatake mountains.

## Discussion

The green-up day was the most important variable (Table 1; Fig. 1) to predict autumn color brightness in our research sites. This result is inconsistent with previous studies reporting that warmer autumn temperature was correlated with autumn red coloration in tall temperate deciduous trees in North America^28,30^. Other papers reported that solar radiation was correlated with the autumn color brightness, as greater solar radiation in open canopies than in closed canopies increased the amount of carotenoids in European *Fagus sylvatica* seedlings^31,37^. Although the green-up day was noted as an important factor for autumn phenological timing^33,34^, its effect on color brightness was not previously assessed. The mechanisms behind spring-autumn phenology connection are still under discussion, but earlier spring leaf-out may cause: (1) earlier autumn senescence if leaf life span is fixed^12,38^; (2) water shortage in the early period of the growing season^39^; (3) damage from spring frost^40^ and insect outbreaks^41^; and (4) earlier achievement of maximum carbon content, which advances senescence in autumn^42^. Although further quantitative analyses comparing the effect of green-up day with the other temperature and radiation on autumn color brightness at the other sites are needed, we think the leaf lifespan matters here. Relating to the previous mechanism (1) and (4), the duration of leaf lifespan is prolonged in early green-up years, resulting in older leaves in autumn. Our data show a positive effect of green-up day on peak autumn color day (peak autumn color day ≈ 0.359 × green-up day, using random intercept for each individual detected by the area of interest (AOI)), associating an early green-up day with an early peak autumn color day, but this autumn earliness was weaker than spring earliness (green-up day) (the slope parameter (0.359) was lower than 1), resulting in older leaves in an early green-up year. This early autumn leaf senescence in an early spring year can be listed as a main cause of the correlation between autumn color brightness and green-up day in Japanese alpine regions. Older leaves tend to have decreased physiological performance, possibly reducing the biosynthesis of pigments^43,44^. This prolonged leaf duration effect would be more serious in less productive alpine zones than in warmer regions, due to the limited resources in alpine zones for creating enough defensive and adaptive physiological mechanisms.

Future reduction of alpine autumn color is evident in warmer lower-elevation or latitude sites (Fig. 4), suggesting these areas as vulnerable to a reduction of autumn color brightness. Regions whose current annual mean temperature is > 3.5 °C were suggested to expect a severe VARI decrease (Fig. 4). These hot regions would be in or near cool temperate regions (below alpine zone) according to the species distribution model of Japanese stone pine (*Pinus pumila*), a typical alpine species, for which the warmth index (Sum of (Tm-5) when Tm is above 5 °C: (Tm: Monthly Mean Temperature)) below 43.2 °C month (i.e., an annual mean temperature of ~ 4 °C according to the mesh climate Japan 2010 dataset) is the first requirement of its distribution^36^. This low-elevation alpine plant distribution^45^ is possibly a result of stress conditions caused by volcanic activity^46^ heavy snowfall^47^ cool microsites such as wind-hole cool spots^48^ or algific talus^49^. Our projection area included these special areas because of their vegetation structure and component species, but caution is needed because some of this low-elevation alpine vegetation would not contain our target species (*S. matsumurana*, *B. ermanii*, and *A. tschonoskii*).

Although the projected color change has wide uncertainty (i.e., VARI decrease of up to 25%) according to the predicted rate of warming among GCMs and RCPs (Fig. 3), the trend of color decrease was consistent. Such a decrease would cause widespread economic loss in the tourism industry. However, this prediction contradicts previous work predicting increases in economic income by Chinese tourists in Japanese temperate regions where a later autumn leaf color season will coincide with Chinese national holidays^15^. Although these authors assessed economic effects by focusing on color timing, it is clear that color brightness is also relevant. In any case, our results demonstrate that phenological perspectives alone are not enough in assessing alpine autumn leaf color tourism, and future changes in autumn color brightness should be considered for this important cultural ecosystem service.

Future reduction of autumn color brightness could also affect ecosystem productivity or the C: N balance, although linkage to a mechanistic process-based vegetation model (especially for alpine vegetation, e.g^50,51^. is needed for its quantitative assessment. The home-field advantage of the decomposing process^52^ should amplify the complexity of ecosystems through spatiotemporal matching of decomposing ability and litter quality and quantity, especially when climate change and related vegetation shifts occur. Our work is a macro-scale, simple projection, but there is potential to connect and broaden research on ecosystem functions.

Our focus on macro-scale national patterns could lead to mismatches in fine-scale predictions. The site cameras (Supplementary Fig. S5) cover limited slope direction and topography. Snow cover tends to differ with slope direction and topography^53,54^ so our projections have uncertainties in relation to these landscape micro-patterns. Local management plans need future fine-scale analyses and projections. Another limitation is local adaptation or phenotypic plasticity on physiological performance^55,56^. The spatial extent of our observation data is limited, then it possibly causes uncertainty, especially for the low-elevation alpine zones. Future modeling should focus on finer spatial scales. This detailed research on individual differences (e.g., phenotype, micro environment, age, etc.) can increase the model accuracy, which is another limitation of our model 5 (R^2^ < 0.5, Table 1). Although we focused on several typical Japanese alpine species, future climate change has the possibility of causing compositional changes^57^. This ecological effect of competitions and distributions should be mentioned further.

## Conclusion

Previous phenological studies focused on the timing of autumn leaf coloring. A predicted timing match with holiday seasons under future climate conditions gives a predicted increase in economic income from tourism^15^. However, our predicted decrease in autumn leaf color brightness rather suggests a possibility of decreasing income. More attention to autumn leaf color brightness is needed, for which our broad-scale modeling can be a first step toward revealing the underlying mechanisms. Future work should approach the finer-scale ecophysiological mechanisms to support forest management under future climate conditions. Fine-scale modeling can be used to predict refugia^58^ for bright autumn colors under future conditions, which should be a target area for conservation or tourism use. It might be possible to artificially mimic and manipulate such environments (e.g., by accumulating snow in specific valleys) to retain bright autumn vistas, although careful ethical discussion is needed, as human interventions could affect the integrity of these largely unspoiled alpine regions. Approaches to mechanisms of local adaptation and assisted gene flow from populations in warmer locations^59,60^ should be considered to stabilize autumn color brightness, although ethical discussion is needed for this approach too. For such analyses of ecophysiological mechanisms of autumn leaf color, broader observations on a finer spatial scale are crucial, especially in alpine vegetation. Potential data sources include fine-scale remote-sensing data from satellites (e.g., Dove satellite constellation) and drones^61^ civil science-based observation networks or analysis of data from social network services^62^ and an increase in repeated camera observations in collaboration with mountain huts or park rangers^63^. We hope that this will stimulate such future works.

## Materials and methods

Overall flowchart of our analyses was summarized in Supplementary Fig. S6.

### Repeated camera observations

We used digital repeat photography data captured at three alpine sites in Japan (details are in Koide, et al.^35^: Asahidake (ASH, 43°39′44″N, 142°49′31″E, 1600 m a.s.l.), in Hokkaido, and two sites in the central alpine area of Honshu, Murodo (MRD, 36°34′40″N, 137°36′04″E, 2450 m a.s.l.), on the Sea of Japan side, and Gokurakudaira (GOK, 35°46′39″N, 137°48′47″E, 2700 m a.s.l.), on the Pacific Ocean side (Supplementary Fig. S5). We downloaded the data for ASH (2016–2020) from the Internet Nature Institute (https://www.sizenken.biodic.go.jp/index_en.php, accessed on 2025/5/7). For GOK (2012–2016) and MRD (2011–2016), we used original high-resolution photographs from the Center for Global Environmental Research (https://db.cger.nies.go.jp/gem/ja/mountain/, accessed on 2025/5/7; note that the resolution is reduced). We used different year range for ASH because the camera and angle settings changed in June 2016 in this site. Camera manufacturer and model were Panasonic BB-SW174WA for ASH, Nikon D7000 for GOK, and Canon EOS 5D Mk2 for MRD. The mean annual temperature at the sites ranges from − 1.8 to + 0.1 °C (ASH: −1.8, GOK: +0.1, MRD: −0.4) and annual precipitation from 1591 to 2714 mm (ASH: 1591.3, GOK: 2714.1, MRD: 2288.7) (1981–2010 average) according to 1-km-mesh climate data^64^. All sites have alpine vegetation in a mosaic of several dominant species, including *Pinus pumila*, *Sorbus matsumurana*, *Betula ermanii*, and *Acer tschonoskii*. The digital photographs were taken hourly; photographs captured around noon were selected in consideration of sunshine and the slope direction of each site (ASH: 12:00–14:00, GOK: 9:00–16:00, MRD: 10:00–14:00). Ten areas of interest (AOIs) were set (Supplementary Fig. S5), and average values of each of the red, green, and blue channels in each AOI were calculated. We calculated the visible atmospherically resistant index (VARI) as an indicator of the leaf color brightness as VARI = (Red − Green)/(Red + Green − Blue). Note that the numerator was converted from the ordinal VARI index (i.e. Green − Red) to focus on red. We used VARI for its relatively robust performance at explaining autumn leaf color brightness compared to other indices^35^. We used the maximum VARI value of each AOI in each year as the autumn color brightness in the analyses. The images were processed in the free scripting language ZeScript and the free ZeGraph software (http://www.zegraph.com/). To focus on autumn leaf coloring, we selected photos from 1 September to the first snowfall of the season (early to mid-October) for analysis. The number of photographs for each year was around 183, 315, and 250 for ASH, GOK, and MRD, respectively. At ASH, all AOIs were set in red leaf areas. At GOK and MRD, five and six AOIs, respectively, were set in red leaf areas, and the others were set in yellow leaf areas. *Sorbus matsumurana* is the main red-leafed species at our sites, although *Sorbus sambucifolia* may also have been present in the AOIs. *Betula ermanii* and *Acer tschonoskii* are the main yellow-leafed species.

To detect the effects by spring phenology, we detected the green-up day and the snowmelt day from spring camera observation data (May–July) from each AOI. The snowmelt day was detected by eye, because automated detection misidentifies by observing snow covers behind the vegetation through gaps in bare branches. The green-up day was detected by fitting a sigmoid curve to the seasonal changes in the green ratio (GR) index (GR = Green/(Red + Green + Blue)). The day when the sigmoid-fitted GR growth rate first reached the maximum was selected as the green-up day according to the previous work^63^.

### Climate data

Past climatic data (Supplementary Table S1) were gathered from on-site observations or the nearest AMeDAS (Automated Meteorological Data Acquisition System) weather station of the Japan Meteorological Agency (JMA; https://www.data.jma.go.jp/stats/etrn/index.php, accessed on 2025/5/7). Daily temperature, precipitation, sunshine duration, and snow depth data were collected. At GOK (35°46′, 137°48′, 2630 m a.s.l.), we collected hourly temperature data with a HD9817T1R sensor (DeltaOHM; http://ims.shinshu-u.ac.jp/~metims_web/index.php?sokuhou, accessed on 2025/5/7). At MRD, we used temperature data provided by the Tateyama-Murodou mountain villa (http://www.murodou.co.jp/kishou/test.php, accessed on 2025/5/7). For other climatic data, the nearest AMeDAS stations around GOK (the average among Kiso-Fukushima (35°50.4′N, 137°41.3′E, 750 m a.s.l), Ina (35°49.5′N, 137°57.3′E, 633 m a.s.l.), Iijima (35°39.2′N, 137°53.9′E, 728 m a.s.l.) for most variables, and Kaida-Kougen (35°56.3′N, 137°36.1′E, 1130 m a.s.l.) for the maximum snow depth) and MRD (the average among Omachi (36°31.4′N, 137°49.9′E, 784 m a.s.l.) and Kamiiichi (36°40.2′N, 137°25.4′E, 296 m a.s.l.) for most variables, and Omachi for the maximum snow depth) was used. At ASH, we collected temperature and other data from the nearest AMeDAS stations (Shibinai (43°38.6′N, 142°34.9′E, 310 m a.s.l.) for most variables, and Sounkyo (43°45.2′N, 142°55.8′E, 540 m a.s.l.) for the maximum snow depth). Since the elevational range among AOIs at GOK and MRD is wide, we accounted for the elevational temperature difference by using a lapse rate of 0.55 °C/100 m^65^. We collected global solar radiation data from the Agro-Meteorological Grid Square Data (AMGSD) of the National Agriculture and Food Research Organization (https://amu.rd.naro.go.jp/wiki_open/doku.php?id=start2, accessed on 2025/5/7). This dataset is spatially interpolated at a 1-km resolution through statistical modeling. In projecting the spatial distribution of autumn leaf color brightness, we used the AMGSD as current climatic data.

We used climatic projection data of the World Climate Research Program’s Coupled Model Intercomparison Project Phase 5 as future climate data. We used the Social Implementation Program on Climate Change Adaptation Technology (SI-CAT: https://research-er.jp/categories/4160, accessed on 2025/5/7) to statistically downscale the original global climate models (GCMs) to a 1-km scale. We used two GCMs (MIROC5, MRI-CGCM3), two representative concentration pathway scenarios (RCP 2.6, 8.5), and two time-periods (2031–2050, 2081–2100) to test future climatic uncertainties. The two GCMs were made by Japanese research institutes, and MIROC5 shows a higher temperature warming trend (Fig. S3). The baseline condition was set at 1981–2000. The bias in each 1-km cell was corrected first by calculating temperature differences and the ratio of precipitation between current (1981–2000) and future time-periods in the downscaled GCMs, and then by adding the temperature difference to or by multiplying the ratio of precipitation difference by the current AMGSD data.

### Prediction of snowmelt day

To estimate future changes in green-up days, we focused on the snowmelt day, which has a strong correlation with the green-up day (Supplementary Fig. S2). We based a snow projection model on the degree-day method^66^ as used to project future distribution changes among Japanese deer^67^. The daily existence of snow cover is predicted from the mean temperature and precipitation in 1-km cells. The snowmelt coefficient was optimized by using SPOT Vegetation satellite data^68^. From the predicted daily snow existence in the present and future periods, we set the snowmelt day as the day following the last snow day. Then we predicted the current and future green-up days from the snowmelt day with the equation relating them (Supplementary Fig. S2).

### Analysis

To predict the annual maximum VARI in each AOI, we constructed linear mixed models for every combination among 14 explanatory variables (Supplementary Table S1). The explanatory variables included monthly variables in each July and August (the productive period for alpine vegetation without snow cover). The broader seasonal range (June to September) was also analyzed in the same way, but the results were almost the same (Supplementary Table S2), then we report results in July to August in the main text. We used temperature (mean/maximum/minimum)^28^^[–30^, global solar radiation and total sunshine duration^31,37,69^ total precipitation^28^ maximum snow depth^70^ and the annual green-up day^33,34,71,72^ for their effects on autumn phenology and color amount. We set leaf color type (red/yellow) for each AOI as a fixed parameter, because yellows have lower VARI values than reds. Since our model treats the VARI difference between red and yellow coloring as constant (Fig. 1), we only showed predictions for the red coloring in our maps and graphs. Each AOI was used as a random intercept, as this analysis focused on temporal patterns at each AOI to predict future changes, given the limited climatic and topographic research range of our dataset. All possible combinations among explanatory variables were compared using Akaike’s information criteria (AIC). Models with significant correlations (*p* < 0.05) among explanatory variables were removed.

## Supplementary Information

Below is the link to the electronic supplementary material.

Supplementary Material 1

Supplementary Material 2

## Data Availability

The datasets generated and analyzed during the current study are provided in the Supplementary Information.

## Declarations

### Reference to pre-print servers

The Preprint version of this article is available in the SSRN server, [https://ssrn.com/abstract=4980369]

## References

[1] Fox, N. et al. A novel data source for cultural ecosystem service studies. *Ecosyst. Serv.* **50**, 101331. 10.1016/j.ecoser.2021.101331 (2021).

[2] Hernandez-Morcillo, M., Plieninger, T. & Bieling, C. An empirical review of cultural ecosystem service indicators. *Ecol. Indic.* **29**, 434–444. 10.1016/j.ecolind.2013.01.013 (2013).

[3] Ge, Q. S., Dai, J. H., Liu, J., Zhong, S. Y. & Liu, H. L. The effect of climate change on the fall foliage vacation in China. *Tour Manag*. **38**, 80–84. 10.1016/j.tourman.2013.02.020 (2013).

[4] Hall, C. M., Scott, D. & Gossling, S. Forests, climate change and tourism. *J. Herit. Tour*. **6**, 353–363. 10.1080/1743873x.2011.620252 (2011).

[5] Yang, J., Wang, X. R. & Zhao, Y. Leaf color attributes of urban colored-leaf plants. *Open. Geosci.* **14**, 1591–1605. 10.1515/geo-2022-0433 (2022).

[6] Zhang, Z. et al. Relationship between forest color characteristics and scenic beauty: case study analyzing pictures of mountainous forests at sloped positions in Jiuzhai valley, China. *Forests* **8**, 63. 10.3390/f8030063 (2017).

[7] Fu, Y. S. H. et al. Larger temperature response of autumn leaf senescence than spring leaf-out phenology. *Glob Chang. Biol.* **24**, 2159–2168. 10.1111/gcb.14021 (2018). 29245174 10.1111/gcb.14021

[8] Heide, O. M. & Prestrud, A. K. Low temperature, but not photoperiod, controls growth cessation and dormancy induction and release in Apple and Pear. *Tree Physiol.* **25**, 109–114. 10.1093/treephys/25.1.109 (2005). 15519992 10.1093/treephys/25.1.109

[9] Liu, G. H., Chen, X. Q., Zhang, Q. H., Lang, W. G. & Delpierre, N. Antagonistic effects of growing season and autumn temperatures on the timing of leaf coloration in winter deciduous trees. *Glob Chang. Biol.* **24**, 3537–3545. 10.1111/gcb.14095 (2018). 29460318 10.1111/gcb.14095

[10] Fracheboud, Y. et al. The control of autumn senescence in European Aspen. *Plant. Physiol.* **149**, 1982–1991. 10.1104/pp.108.133249 (2009). 19201914 10.1104/pp.108.133249 PMC2663763

[11] Soolanayakanahally, R. Y., Guy, R. D., Silim, S. N. & Song, M. H. Timing of photoperiodic competency causes phenological mismatch in Balsam Poplar (*Populus Balsamifera* L). *Plant. Cell. Environ.* **36**, 116–127. 10.1111/j.1365-3040.2012.02560.x (2013). 22702736 10.1111/j.1365-3040.2012.02560.x

[12] Lim, P. O., Kim, H. J. & Nam, H. G. Leaf senescence. *Annu. Rev. Plant. Biol.* **58**, 115–136. 10.1146/annurev.arplant.57.032905.105316 (2007). 17177638 10.1146/annurev.arplant.57.032905.105316

[13] Wang, L., Ning, Z. Z., Wang, H. J. & Ge, Q. S. Impact of climate variability on flowering phenology and its implications for the schedule of blossom festivals. *Sustainability* **9**, 1127. 10.3390/su9071127 (2017).

[14] Vitasse, Y. et al. Assessing the effects of climate change on the phenology of European temperate trees. *Agr For. Meteorol.* **151**, 969–980. 10.1016/j.agrformet.2011.03.003 (2011).

[15] Liu, J., Cheng, H., Jiang, D. & Huang, L. Impact of climate-related changes to the timing of autumn foliage colouration on tourism in Japan. *Tour Manag*. **70**, 262–272. 10.1016/j.tourman.2018.08.021 (2019).

[16] Keenan, T. F. et al. Net carbon uptake has increased through warming-induced changes in temperate forest phenology. *Nat. Clim. Change*. **4**, 598–604. 10.1038/Nclimate2253 (2014).

[17] Vitasse, Y., Porté, A. J., Kremer, A., Michalet, R. & Delzon, S. Responses of canopy duration to temperature changes in four temperate tree species: relative contributions of spring and autumn leaf phenology. *Oecologia* **161**, 187–198. 10.1007/s00442-009-1363-4 (2009). 19449036 10.1007/s00442-009-1363-4

[18] Feild, T. S., Lee, D. W. & Holbrook, N. M. Why leaves turn red in autumn. The role of anthocyanins in senescing leaves of red-osier Dogwood. *Plant. Physiol.* **127**, 566–574. 10.1104/pp.127.2.566 (2001). 11598230 PMC125091

[19] Gomez-Sagasti, M. T. et al. Carotenoids and their derivatives: A Swiss army knife-like multifunctional tool for fine-tuning plant-environment interactions. *Environ. Exp. Bot.* **207**, 105229. 10.1016/j.envexpbot.2023.105229 (2023).

[20] Liang, J. & He, J. X. Protective role of anthocyanins in plants under low nitrogen stress. *Biochem. Bioph Res. Co.* **498**, 946–953. 10.1016/j.bbrc.2018.03.087 (2018). 10.1016/j.bbrc.2018.03.087 29548824

[21] Patoine, G. et al. Tree litter functional diversity and nitrogen concentration enhance litter decomposition via changes in earthworm communities. *Ecol. Evol.* **10**, 6752–6768. 10.1002/ece3.6474 (2020). 32724548 10.1002/ece3.6474 PMC7381558

[22] Wang, Y. Z., Zheng, J. Q., Boyd, S. E., Xu, Z. H. & Zhou, Q. X. Effects of litter quality and quantity on chemical changes during Eucalyptus litter decomposition in subtropical Australia. *Plant. Soil.* **442**, 65–78. 10.1007/s11104-019-04162-2 (2019).

[23] Sun, L. J., Ataka, M., Kominami, Y., Yoshimura, K. & Kitayama, K. A constant microbial C/N ratio mediates the microbial nitrogen mineralization induced by root exudation among four co-existing canopy species. *Rhizosphere* **17**, 100317. 10.1016/j.rhisph.2021.100317 (2021).

[24] Luyssaert, S. et al. The European carbon balance. Part 3: forests. *Glob Chang. Biol.* **16**, 1429–1450. 10.1111/j.1365-2486.2009.02056.x (2010).

[25] Rennenberg, H. et al. Nitrogen balance in forest soils: nutritional limitation of plants under climate change stresses. *Plant. Biol.* **11**, 4–23. 10.1111/j.1438-8677.2009.00241.x (2009). 19778364 10.1111/j.1438-8677.2009.00241.x

[26] Gallinat, A. S., Primack, R. B. & Wagner, D. L. Autumn, the neglected season in climate change research. *Trends Ecol. Evol.* **30**, 169–176. 10.1016/j.tree.2015.01.004 (2015). 25662784 10.1016/j.tree.2015.01.004

[27] Junker, L. V. & Ensminger, I. Relationship between leaf optical properties, chlorophyll fluorescence and pigment changes in senescing *Acer saccharum* leaves. *Tree Physiol.* **36**, 694–711. 10.1093/treephys/tpv148 (2016). 26928514 10.1093/treephys/tpv148

[28] Archetti, M., Richardson, A. D., O’Keefe, J. & Delpierre, N. Predicting climate change impacts on the amount and duration of autumn colors in a new England forest. *Plos One*. **8**, e57373. 10.1371/journal.pone.0057373 (2013). 23520468 10.1371/journal.pone.0057373 PMC3592872

[29] Schaberg, P. G., Van den Berg, A. K., Murakami, P. F., Shane, J. B. & Donnelly, J. R. Factors influencing red expression in autumn foliage of sugar maple trees. *Tree Physiol.* **23**, 325–333. 10.1093/treephys/23.5.325 (2003). 12615547 10.1093/treephys/23.5.325

[30] Xie, Y. Y., Civco, D. L. & Silander, J. A. Species-specific spring and autumn leaf phenology captured by time-lapse digital cameras. *Ecosphere* **9**, e02089. 10.1002/ecs2.2089 (2018).

[31] Herbinger, K. et al. Gas exchange and antioxidative compounds in young Beech trees under free-air Ozone exposure and comparisons to adult trees. *Plant. Biol.* **9**, 288–297. 10.1055/s-2006-924660 (2007). 17357021 10.1055/s-2006-924660

[32] Nestola, E. et al. Are optical indices good proxies of seasonal changes in carbon fluxes and stress-related physiological status in a Beech forest? *Sci. Total Environ.* **612**, 1030–1041. 10.1016/j.scitotenv.2017.08.167 (2018). 28892844 10.1016/j.scitotenv.2017.08.167

[33] Fu, Y. Y. et al. Climate and spring phenology effects on autumn phenology in the greater Khingan mountains, Northeastern China. *Remote Sens.* **10**, 449. 10.3390/rs10030449 (2018).

[34] Liu, Q. et al. Delayed autumn phenology in the Northern hemisphere is related to change in both climate and spring phenology. *Glob Chang. Biol.* **22**, 3702–3711. 10.1111/gcb.13311 (2016). 27061925 10.1111/gcb.13311

[35] Koide, D., Ide, R. & Oguma, H. Detection of autumn leaf phenology and color brightness from repeat photography: accurate, robust, and sensitive indexes and modeling under unstable field observations. *Ecol. Indic.* **106**, 105482. 10.1016/j.ecolind.2019.105482 (2019).

[36] Horikawa, M., Tsuyama, I., Matsui, T., Kominami, Y. & Tanaka, N. Assessing the potential impacts of climate change on the alpine habitat suitability of Japanese stone pine (Pinus pumila). *Landsc. Ecol.* **24**, 115–128. 10.1007/s10980-008-9289-5 (2008).

[37] Hansen, U., Fiedler, B. & Rank, B. Variation of pigment composition and antioxidative systems along the canopy light gradient in a mixed beech/oak forest: a comparative study on deciduous tree species differing in shade tolerance. *Trees-Struct Funct.* **16**, 354–364. 10.1007/s00468-002-0163-9 (2002).

[38] Reich, P. B., Walters, M. B. & Ellsworth, D. S. Leaf life-span in relation to leaf, plant, and stand characteristics among diverse ecosystems. *Ecol. Monogr.* **62**, 365–392. 10.2307/2937116 (1992).

[39] Buermann, W., Bikash, P. R., Jung, M., Burn, D. H. & Reichstein, M. Earlier springs decrease peak summer productivity in North American boreal forests. *Environ. Res. Lett.* **8**, 024027. 10.1088/1748-9326/8/2/024027 (2013).

[40] Hufkens, K. et al. Ecological impacts of a widespread Frost event following early spring leaf-out. *Glob Chang. Biol.* **18**, 2365–2377. 10.1111/j.1365-2486.2012.02712.x (2012).

[41] Jepsen, J. U. et al. Rapid Northwards expansion of a forest insect pest attributed to spring phenology matching with sub-Arctic Birch. *Glob Chang. Biol.* **17**, 2071–2083. 10.1111/j.1365-2486.2010.02370.x (2011).

[42] Fu, Y. S. H. et al. Variation in leaf Flushing date influences autumnal senescence and next year’s Flushing date in two temperate tree species. *Proc. Natl. Acad. Sci. U S A*. **111**, 7355–7360. 10.1073/pnas.1321727111 (2014). 24799708 10.1073/pnas.1321727111 PMC4034254

[43] Dhami, N., Tissue, D. T. & Cazzonelli, C. I. Leaf-age dependent response of carotenoid accumulation to elevated CO2 in Arabidopsis. *Arch. Biochem. Biophys.* **647**, 67–75. 10.1016/j.abb.2018.03.034 (2018). 29604257 10.1016/j.abb.2018.03.034

[44] Neill, S. O., Gould, K. S., Kilmartin, P. A., Mitchell, K. A. & Markham, K. R. Antioxidant activities of red versus green leaves in elatostema rugosum. *Plant. Cell. Environ.* **25**, 539–547. 10.1046/j.1365-3040.2002.00837.x (2002).

[45] Wakui, A. & Kudo, G. Ecotypic differentiation of a circumpolar Arctic-alpine species at mid-latitudes: variations in the ploidy level and reproductive system of *Vaccinium vitis-idaea*. *Aob Plants*. **13**, plab015. 10.1093/aobpla/plab015 (2021). 34007436 10.1093/aobpla/plab015 PMC8114225

[46] Leuschner, C. Timberline and alpine vegetation on the tropical and warm-temperate oceanic Islands of the world: elevation, structure and floristics. *Vegetatio* **123**, 193–206. 10.1007/Bf00118271 (1996).

[47] Gansert, D. Treelines of the Japanese Alps - altitudinal distribution and species composition under contrasting winter climates. *Flora* **199**, 143–156. 10.1078/0367-2530-00143 (2004).

[48] Shimokawabe, A. et al. The distribution of cool spots as microrefugia in a mountainous area. *Plos One*. **10**, e0135732. 10.1371/journal.pone.0135732 (2015). 26285206 10.1371/journal.pone.0135732 PMC4540282

[49] Wakui, A. et al. Environmental factors determining the distribution of Highland plants at low-altitude algific talus sites. *Ecol. Res.* **32**, 183–191. 10.1007/s11284-016-1429-9 (2017).

[50] Alekhya, V., Pujar, G. S., Jha, C. S. & Dadhwal, V. K. Simulation of vegetation dynamics in himalaya using dynamic global vegetation model. *Trop. Ecol.* **56**, 219–231 (2015).

[51] Leuzinger, S., Manusch, C., Bugmann, H. & Wolf, A. A sink-limited growth model improves biomass Estimation along boreal and alpine tree lines. *Glob Ecol. Biogeogr.* **22**, 924–932. 10.1111/geb.12047 (2013).

[52] Ayres, E. et al. Home-field advantage accelerates leaf litter decomposition in forests. *Soil. Biol. Biochem.* **41**, 606–610. 10.1016/j.soilbio.2008.12.022 (2009).

[53] Fujihara, Y. et al. Influence of topography and forest characteristics on snow distributions in a forested catchment. *J. Hydrol.* **546**, 289–298. 10.1016/j.jhydrol.2017.01.021 (2017).

[54] Jost, G., Weiler, M., Gluns, D. R. & Alila, Y. The influence of forest and topography on snow accumulation and melt at the watershed-scale. *J. Hydrol.* **347**, 101–115. 10.1016/j.jhydrol.2007.09.006 (2007).

[55] Kijowska-Oberc, J., Staszak, A. M., Kaminski, J. & Ratajczak, E. Adaptation of forest trees to rapidly changing climate. *Forests* **11**, 123. 10.3390/f11020123 (2020).

[56] Vitasse, Y. et al. Genetic vs. non-genetic responses of leaf morphology and growth to elevation in temperate tree species. *Funct. Ecol.* **28**, 243–252. 10.1111/1365-2435.12161 (2014).

[57] Amagai, Y., Kudo, G. & Sato, K. Changes in alpine plant communities under climate change: dynamics of snow-meadow vegetation in Northern Japan over the last 40 years. *Appl. Veg. Sci.* **21**, 561–571. 10.1111/avsc.12387 (2018).

[58] Ackerly, D. D. et al. Topoclimates, refugia, and biotic responses to climate change. *Front. Ecol. Environ.* **18**, 288–296. 10.1002/fee.2204 (2020).

[59] Aitken, S. N. & Bemmels, J. B. Time to get moving: assisted gene flow of forest trees. *Evol. Appl.* **9**, 271–290. 10.1111/eva.12293 (2016). 27087852 10.1111/eva.12293 PMC4780373

[60] Aitken, S. N. & Whitlock, M. C. Assisted gene flow to facilitate local adaptation to climate change. *Annu. Rev. Ecol. Evol. S*. **44**, 367–388. 10.1146/annurev-ecolsys-110512-135747 (2013).

[61] Gong, Z., Ge, W. Y., Guo, J. Q. & Liu, J. C. Satellite remote sensing of vegetation phenology: progress, challenges, and opportunities. *Isprs J. Photogramm*. **217**, 149–164. 10.1016/j.isprsjprs.2024.08.011 (2024).

[62] Nagai, S., Saitoh, T. M. & Miura, T. Peak autumn leaf colouring along latitudinal and elevational gradients in Japan evaluated with online phenological data. *Int. J. Biometeorol.* **64**, 1743–1754. 10.1007/s00484-020-01953-6 (2020). 32562042 10.1007/s00484-020-01953-6

[63] Ide, R. & Oguma, H. A cost-effective monitoring method using digital time-lapse cameras for detecting Temporal and Spatial variations of snowmelt and vegetation phenology in alpine ecosystems. *Ecol. Inf.* **16**, 25–34. 10.1016/j.ecoinf.2013.04.003 (2013).

[64] JMA (Japan Meteorological Agency). *Climate Normals for Japan* (Japan Meteorological Business Support Center, 2012).

[65] Ueno, K. et al. Data archive of meteorological data created through the Japanese alps inter-university cooperative project. *J. Geogr-Tokyo*. **122**, 638–650. 10.5026/jgeography.122.638 (2013).

[66] Kominami, Y., Tanaka, N., Endo, Y. & Niwano, S. Estimation of snow distribution under global warming using data from remote weather stations (AMeDAS). *J. Agric. Meteorol.* **60**, 445–450 (2005).

[67] Ohashi, H. et al. Land abandonment and changes in snow cover period accelerate range expansions of Sika deer. *Ecol. Evol.* **6**, 7763–7775. 10.1002/ece3.2514 (2016). 30128126 10.1002/ece3.2514 PMC6093158

[68] Asaoka, Y. & Kominami, Y. Spatial snowfall distribution in mountainous areas estimated with a snow model and satellite remote sensing. *Hydrol. Res. Lett.* **6**, 1–6. 10.3178/hrl.6.1 (2012).

[69] Tan, X. et al. Estimation of leaf color variances of Cotinus Coggygria based on geographic and environmental variables. *J. Forestry Res.* **32**, 609–622. 10.1007/s11676-020-01118-6 (2021).

[70] Gehrmann, F., Ziegler, C. & Cooper, E. J. Onset of autumn senescence in high Arctic plants shows similar patterns in natural and experimental snow depth gradients. *Arct. Sci.* **8**, 744–766. 10.1139/as-2020-0044 (2022).

[71] Keenan, T. F. & Richardson, A. D. The timing of autumn senescence is affected by the timing of spring phenology: implications for predictive models. *Glob Chang. Biol.* **21**, 2634–2641. 10.1111/gcb.12890 (2015). 25662890 10.1111/gcb.12890

[72] Lee, S., Jeong, S., Park, C. E. & Kim, J. A simple method of predicting autumn leaf coloring date using machine learning with spring leaf unfolding date. *Asia-Pac J. Atmos. Sci.* **58**, 219–226. 10.1007/s13143-021-00251-4 (2022).

