# Supplementary material for: Autumn leaf color brightness of Japanese alpine vegetation is projected to decrease under future climate change
Source: Sci Rep. 2025 Aug 8;15:29064. doi: 10.1038/s41598-025-14547-2 (PMC12334716; doi:10.1038/s41598-025-14547-2)
Supplement: Supplementary file 2 — Supplementary Material 2 [file 41598_2025_14547_MOESM2_ESM.pdf]

Online supporting materials

Article title

Autumn leaf color brightness of Japanese alpine vegetation is projected to decrease under future climate change

Authors

Dai KOIDE[1]*, Reiko IDE[1], Hiroyuki OGUMA[1,2], Keisuke SUZUKI[2], Haruka OHASHI[3], Yuji KOMINAMI[3]

[1] *National Institute for Environmental Studies, 16-2, Onogawa, Tsukuba, Ibaraki 305-8506, Japan*

[2] *Shinshu University, 3-1-1, Asahi, Matsumoto, Nagano 390-8621, Japan*

[3] *Forestry and Forest Products Research Institute, 1 Matsunosato, Tsukuba, Ibaraki 305-8687, Japan*

***Corresponding author:** Dai KOIDE

Table S1. Climatic predictor variables.

	Variable	Unit	Form	Month	Abbreviation
1	Mean temperature	°C	mean	7	Temp_7
2				8	Temp_8
3	Maximum temperature	°C	max	7	Tmax_7
4				8	Tmax_8
5	Minimum temperature	°C	min	7	Tmin_7
6				8	Tmin_8
7	Global solar radiation	MJ/m^2	sum	7	GSR_7
8				8	GSR_8
9	Sunshine duration	h	sum	7	Sun_7
10				8	Sun_8
11	Precipitation sum	mm	sum	7	Prec_7
12				8	Prec_8
13	Green-up day	–	–	–	Gup
14	Maximum snow depth	cm	max	–	MSD

Table S2. The best 10 model structures and their performances based on broader seasonal (June to September) climate variables.

Model	Structure	AIC	R^2
1	Gup + Temp_7	-130.3	0.51
2	Gup + Temp_6	-129.4	0.60
3	Gup + Prec_8	-126.5	0.51
4	Gup + Temp_6 + Sun_6	-125.8	0.62
5	Gup + Temp_7 + Sun_6	-125.5	0.52
6	Gup + Temp_7 + GSR_8	-124.1	0.51
7	Gup + Prec_8 + Sun_8	-122.8	0.53
8	Gup	-122.2	0.47
9	Gup + Temp_7 + Sun_8	-121.5	0.51
10	Gup + Temp_6 + GSR_8	-121.2	0.61

"_6", June; "_7", July; "_8", August; "_9", September. Gup, green-up day; Temp, mean temperature; Tmax, maximum temperature; Tmin, minimum temperature; Prec, precipitation; GSR, global solar radiation. AIC, Akaike's information criteria.

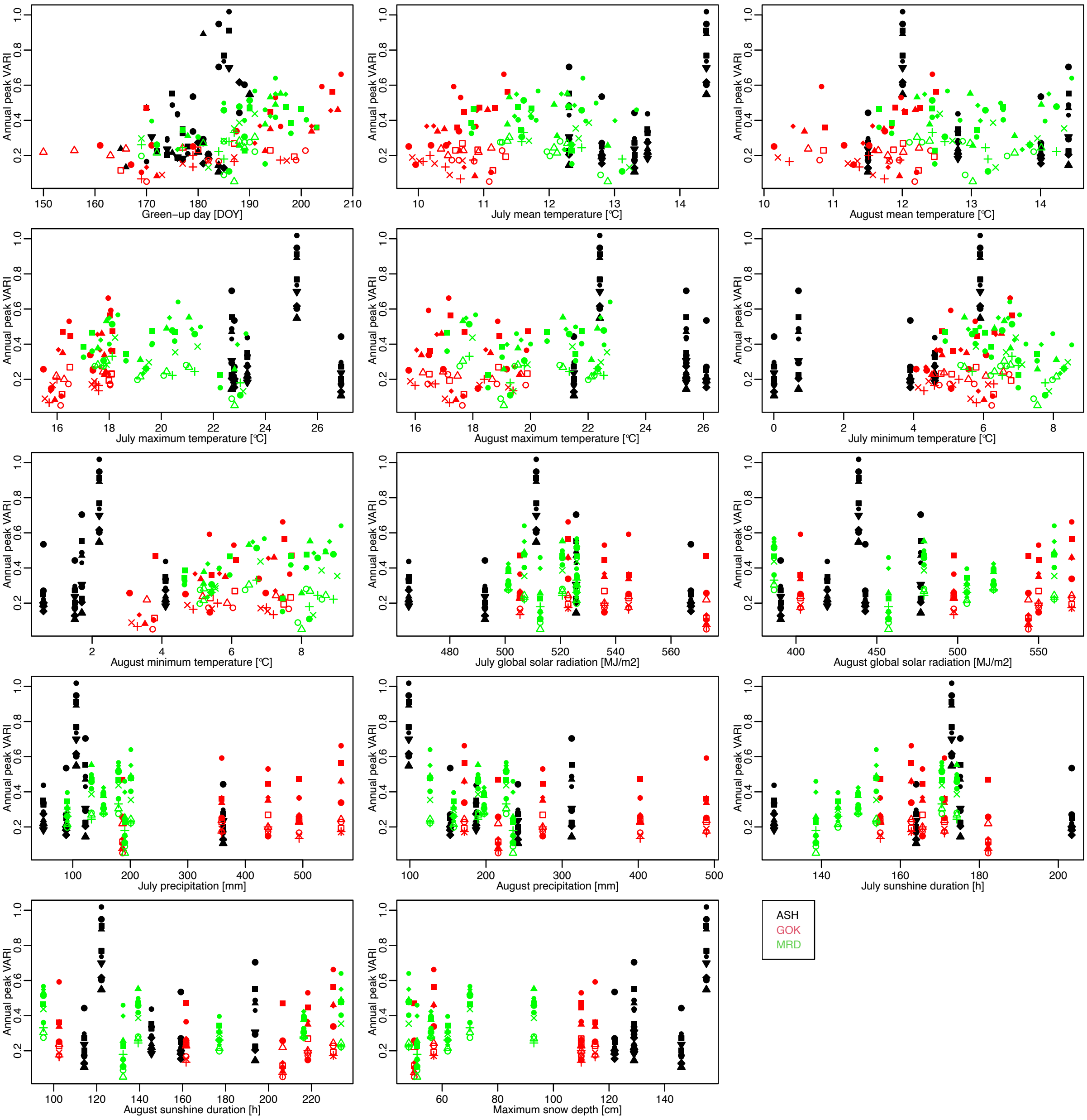

Fig. S1 Scatter plots of annual peak VARI versus 14 explanatory variables. Different symbols represent different areas of interest (AOIs), and filled symbols mean red coloring. Note that for temperature variables in ASH, the elevational range of AOIs was smaller in this site, causing a discrete form only for this site.

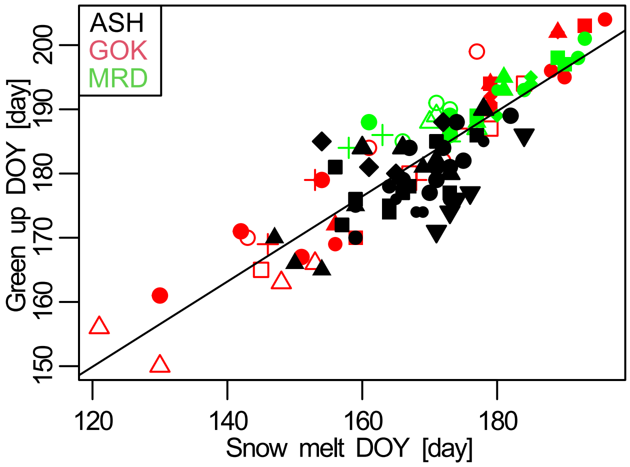

Fig. S2 Relationship between green-up day and snowmelt day (day of year, DOY). Different symbols represent different areas of interest (AOIs). Data were obtained from time-lapse cameras at Asahidake (ASH), Gokurakudaira (GOK), and Murodo (MRD). The regression line by linear model was estimated as Green up DOY = 0.6632 × Snow melt DOY + 70.32 ($P < 0.001$, $r^2 = 0.76$).

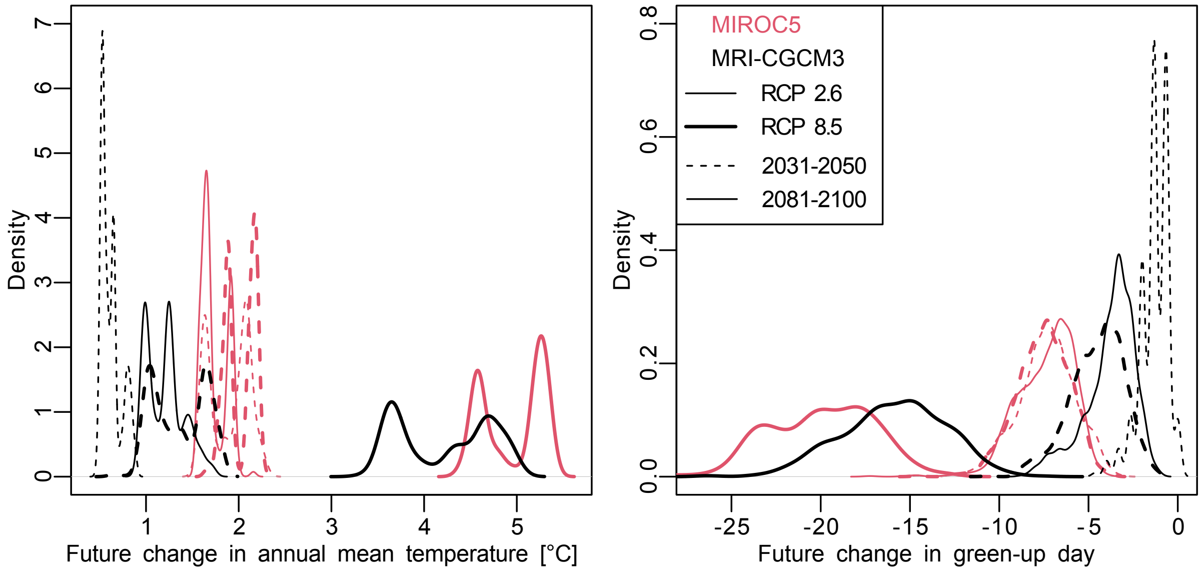

Fig. S3 Density plot of future changes in annual mean temperature and green-up day. The difference between the current and future mean temperature and green-up day was calculated in each alpine vegetation cell and was summarized in a density plot for the national scale. These show future warming and an earlier green-up trend.

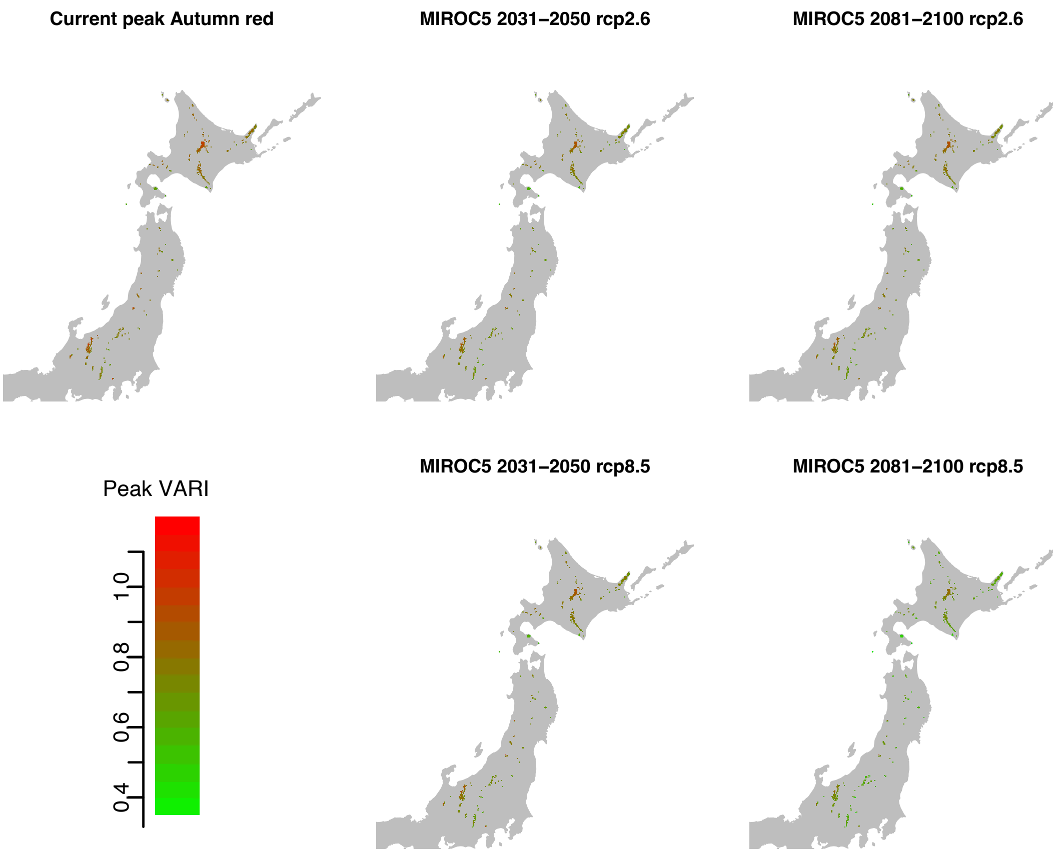

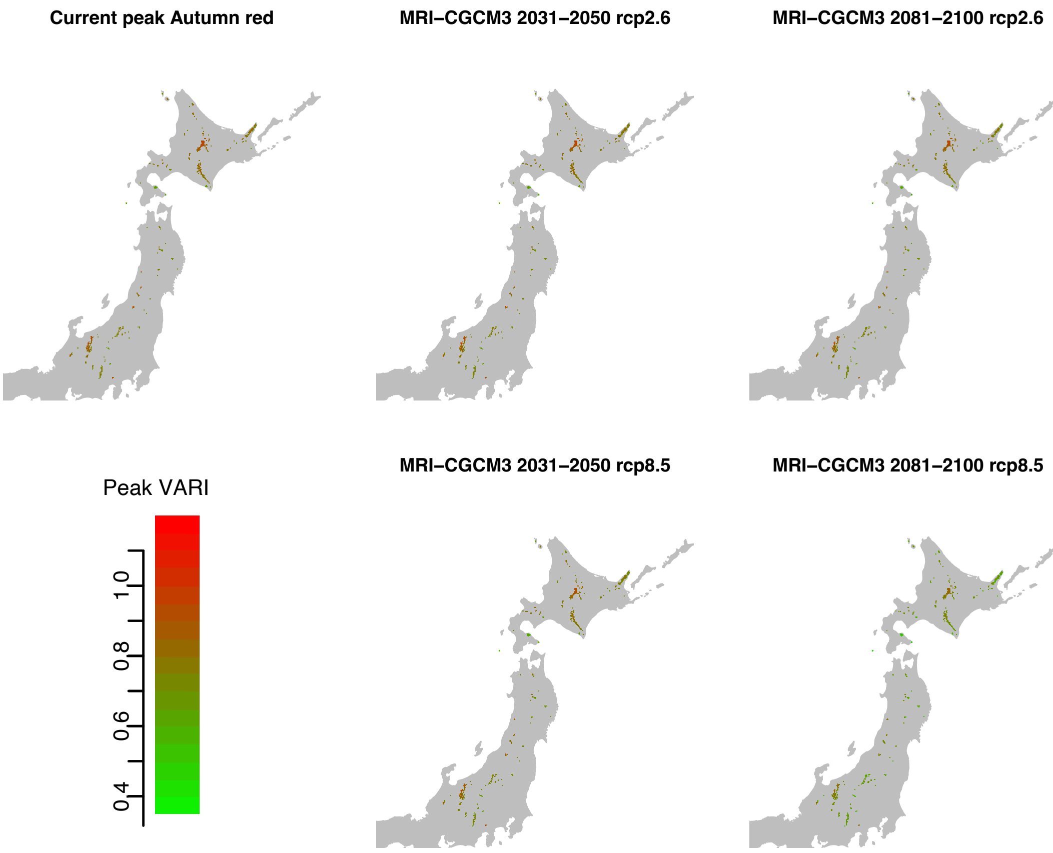

Fig. S4 Map of current and future predicted peak VARI values by each GCM.

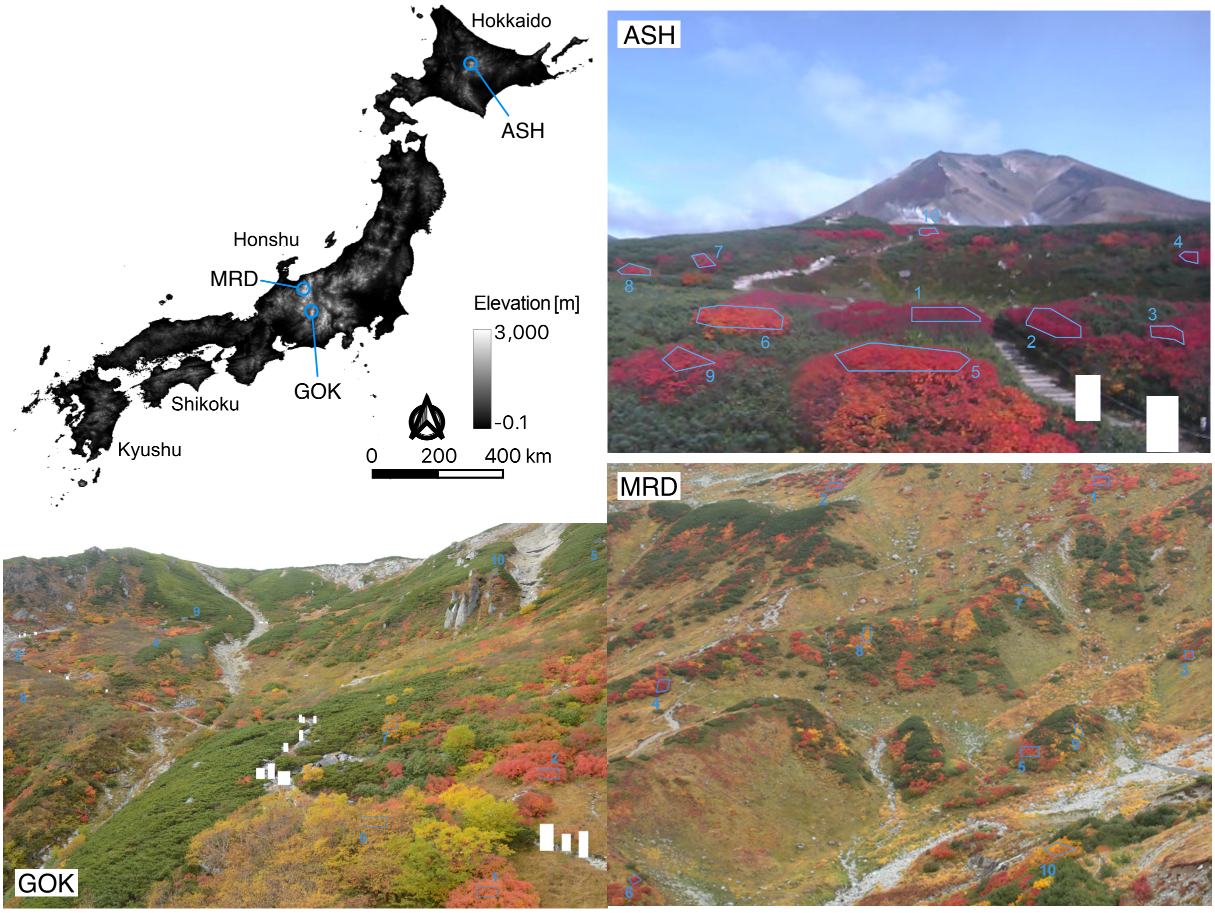

Fig. S5 Areas of interest (AOIs, blue numbered polygons) in each research site image. A detailed description of each site and AOI is reported in Koide et al. (2019), although the number of AOIs at ASH was increased here to match the other sites. The photos are modified from Koide et al. (2019). White rectangles on the photo obscure hikers. Camera angle was set for northeast (ASH), northwest (GOK), and east (MRD) directions. ASH, Asahidake; GOK, Gokurakudaira; MRD, Murodo.

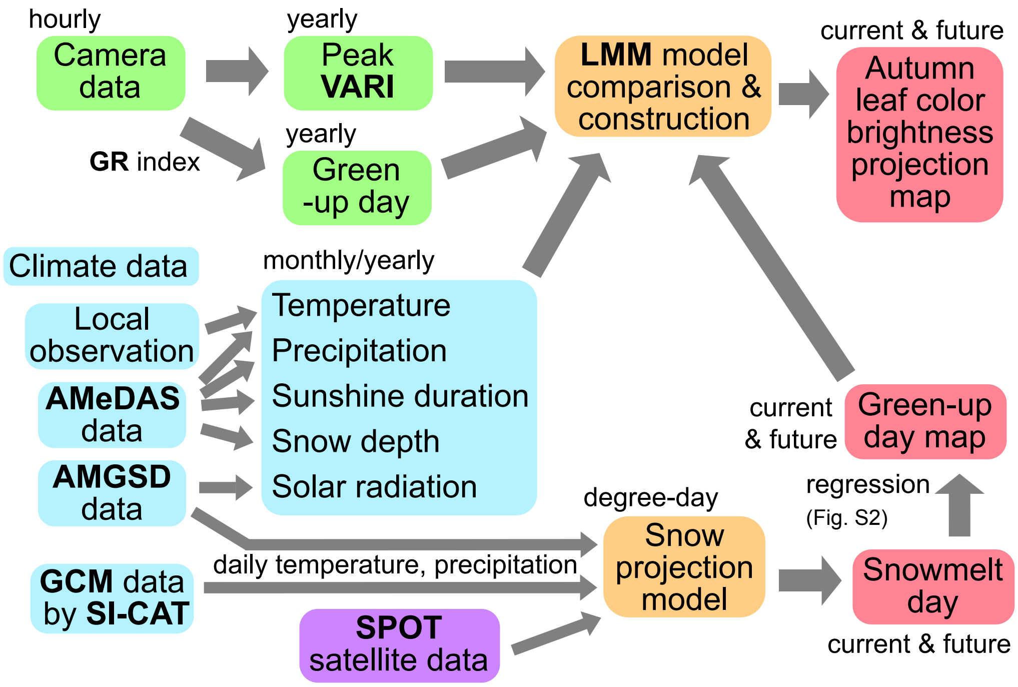

Fig. S6 Flowchart of the overall analyses.
